# Recent Advances for the Direct Introduction of the CF_2_Me Moiety

**DOI:** 10.3389/fchem.2019.00111

**Published:** 2019-03-04

**Authors:** Elodie Carbonnel, Thomas Poisson, Philippe Jubault, Xavier Pannecoucke, Tatiana Besset

**Affiliations:** ^1^Normandie Univ, INSA Rouen, UNIROUEN, CNRS, COBRA (UMR 6014), Rouen, France; ^2^Institut Universitaire de France, Paris, France

**Keywords:** organofluorine chemistry, synthetic methodology, emergent fluorinated groups, CF_2_Me-containing reagent, C-CF_2_Me bond formation

## Abstract

Fluorine-containing molecules are compounds of interest in materials as well as in pharmaceutical and agrochemical industries. Therefore, developments in that research field are tremendous and a special focus was dedicated to the design and the study of emergent fluorinated groups. In particular, the CF_2_Me residue is attractive as it could be used for example as a bioisostere of the methoxy group. Despite the clear asset that represents this fluorinated moiety and in complement to the traditional approaches used to construct the CF_2_Me residue from existing functional groups, the quest for direct methodologies for the 1,1-difluoroethylation reaction of molecules has triggered a strong interest from the scientific community. This Mini-review will focus on the recent advances toward the design of reagents and their applications for the direct 1,1-difluoroethylation of various classes of compounds.

## Introduction

The design of tools and unprecedented synthetic pathways in organofluorine chemistry is of prime importance to extend the current tool box (Landelle et al., 2013; Liang et al., 2013; Besset et al., 2014, 2016; Egami and Sodeoka, 2014; Merino and Nevado, 2014; Belhomme et al., 2015; Champagne et al., 2015; Ni and Hu, 2016; Lemos et al., 2018; Song et al., 2018). With more than 40% of agrochemicals and 25% of pharmaceuticals having at least one fluorine atom, this research field is very active and has witnessed a strong interest from academic and industry laboratories (Purser et al., 2008; Fujiwara and O'Hagan, 2014; Ilardi et al., 2014; Wang et al., 2014; Gillis et al., 2015; Ni et al., 2015; Meanwell, 2018). Taking benefit from the unique features of the fluorine atom and fluorinated groups, the biological and physical properties of a molecule might be modulated at will (O'Hagan, 2008). Therefore, it appeared as crucial to further develop efficient transformations for the introduction of fluorinated moieties onto complex molecules and to design and study new fluorine-containing moieties. Among these emergent fluorinated groups, a special attention was paid to the 1,1-difluoroethyl group. Present in various compounds of interest, the CF_2_Me residue appears as a fluorinated bioisostere of the alkoxy ethers. Indeed, the replacement of the oxygen atom by a CF_2_ residue makes the molecules more metabolically stable and the presence of a CF_2_Me moiety impacts the spatial geometry (difference of conformational preference of an OMe vs. CF_2_Me group) although keeping similar electronic and steric properties (Zhou et al., 2013). In addition, the metabolic stability of the molecules might be modulated by replacing a benzylic methylene residue by a CF_2_ one as it was the case for a urea transporter B (UTB) inhibitor (II), used for edema (Anderson et al., 2012). In addition, these compounds bearing this fluorinated moiety already demonstrated interesting properties, like in the case of edema and malaria treatment, for instance (Coteron et al., 2011; Anderson et al., 2012). Consequently, over the last years, the landscape of this research field has been impacted by key contributions from several research groups, who have pioneered the synthesis and application of original reagents to construct C-CF_2_Me bonds. The synthesis of CF_2_Me-containing molecules mainly relied on the fluorination of (1) carbonyl derivatives (or analogs i.e., thiocarbonyl compounds), (2) benzylic positions, (3) alkynes, or (4) alkenes and was well-documented (for selected examples: Markovskij et al., 1973; Middleton, 1975; York et al., 1996; Lal et al., 1999; Reddy et al., 2005; Yamauchi et al., 2008; Umemoto and Singh, 2012; Xia et al., 2013; Ilchenko et al., 2014; Okoromoba et al., 2014; Xu et al., 2014; Ma et al., 2015; Koperniku et al., 2016; Hua et al., 2017; Li et al., 2017; Zhao et al., 2017; Iacono et al., 2018; Tomita et al., 2018). In sharp contrast, the design of new reagents or methodologies for the direct incorporation of this fluorinated moiety onto molecules is still underdeveloped. Recently Li, Dong et al. reviewed the synthesis of (1,1-difluoroethyl)arenes based on the construction of the CF_2_Me motif and few examples describing its direct incorporation onto arenes were reported (Li et al., 2018).

The aim of this mini-review is to showcase and discuss the recent advances made on novel synthetic strategies for the direct introduction of the emergent CF_2_Me group onto various classes of molecules (arenes, aliphatic, and carbonyl compounds). Therefore, in this review, we will highlight the new technological solutions based on the design of original reagents and methodologies to build up C-CF_2_Me bonds. Note that the approaches employed to construct the CF_2_Me group will not be discussed. Taking these considerations in mind, a first strategy to access to CF_2_Me-containing molecules relies on the introduction of the CF_2_Me group by using nucleophilic reagents, whereas a second approach deals with the construction of a C-CF_2_Me bond according to a radical pathway.

## 1,1-Difluoroethylation of Molecules Using a Nucleophilic CF_2_Me-Containing Reagent

### Transition Metal-Free Reactions to Access CF_2_Me-Containing Molecules

In this section, key advances for the direct 1,1-difluoroethylation of carbonyl derivatives via a transition metal-free process will be depicted.

#### TESCF_2_Me and TMSCF_2_Me as CF_2_Me Sources

**Scheme 1:**
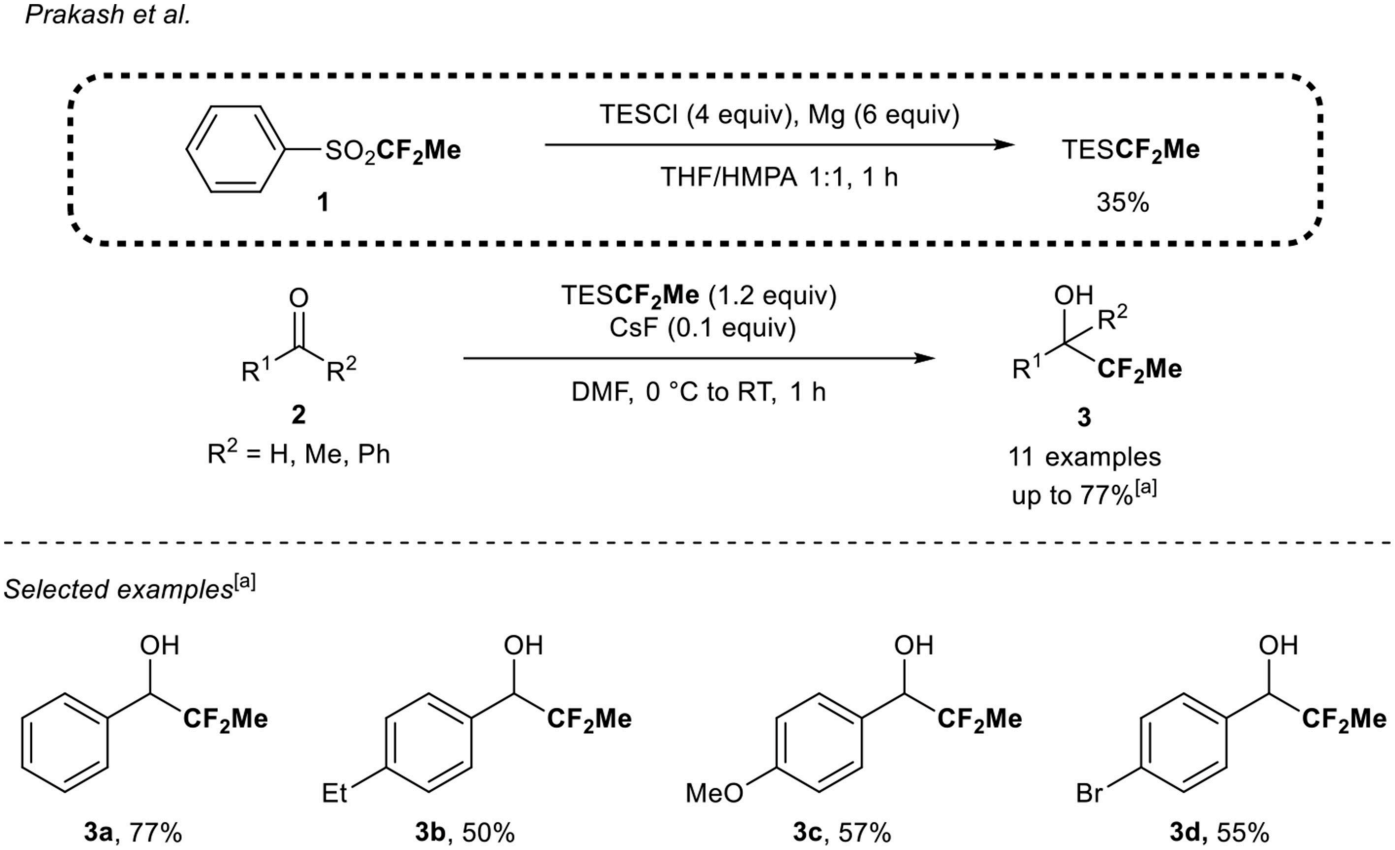
1,1-Difluoroethylation of carbonyl derivatives **2** with the TES**CF**_**2**_**Me** reagent. [a] ^1^H NMR yield of the crude reaction mixture.

In their quest for new fluorine-containing reagents, Prakash et al. (Mogi et al., 2007) reported the synthesis of a 1,1-difluoroethylated reagent, the TESCF_2_Me (Scheme 1). The latter resulted from the reaction between TESCl and (1,1-difluoroethyl)phenylsulfone **1** in the presence of magnesium metal in 35% yield on a gram scale. The precursor **1** was itself prepared in three steps from thiophenol (Langlois, 1988; Mogi et al., 2007). Note that the nature of the solvents (THF/HMPA, 1:1) was crucial to ensure the full conversion of the sulfone **1** into the desired TESCF_2_Me reagent. When this reagent was reacted with aromatic aldehydes **2**, the corresponding 1,1-difluoroethylated secondary alcohols **3** were obtained in moderate to good ^19^F NMR yields (50–77%). The reaction was tolerant to electron-donating groups (**3b,c**) and halogen (**3d**). The reaction was also sensitive to steric hindrance since when the reaction was conducted with a sterically hindered aldehyde or ketone, only traces of products were obtained. Finally, an enolizable aldehyde and ketone and an α,β-unsaturated aldehyde were not suitable substrates in that transformation and constituted the main limitations.

**Scheme 2:**
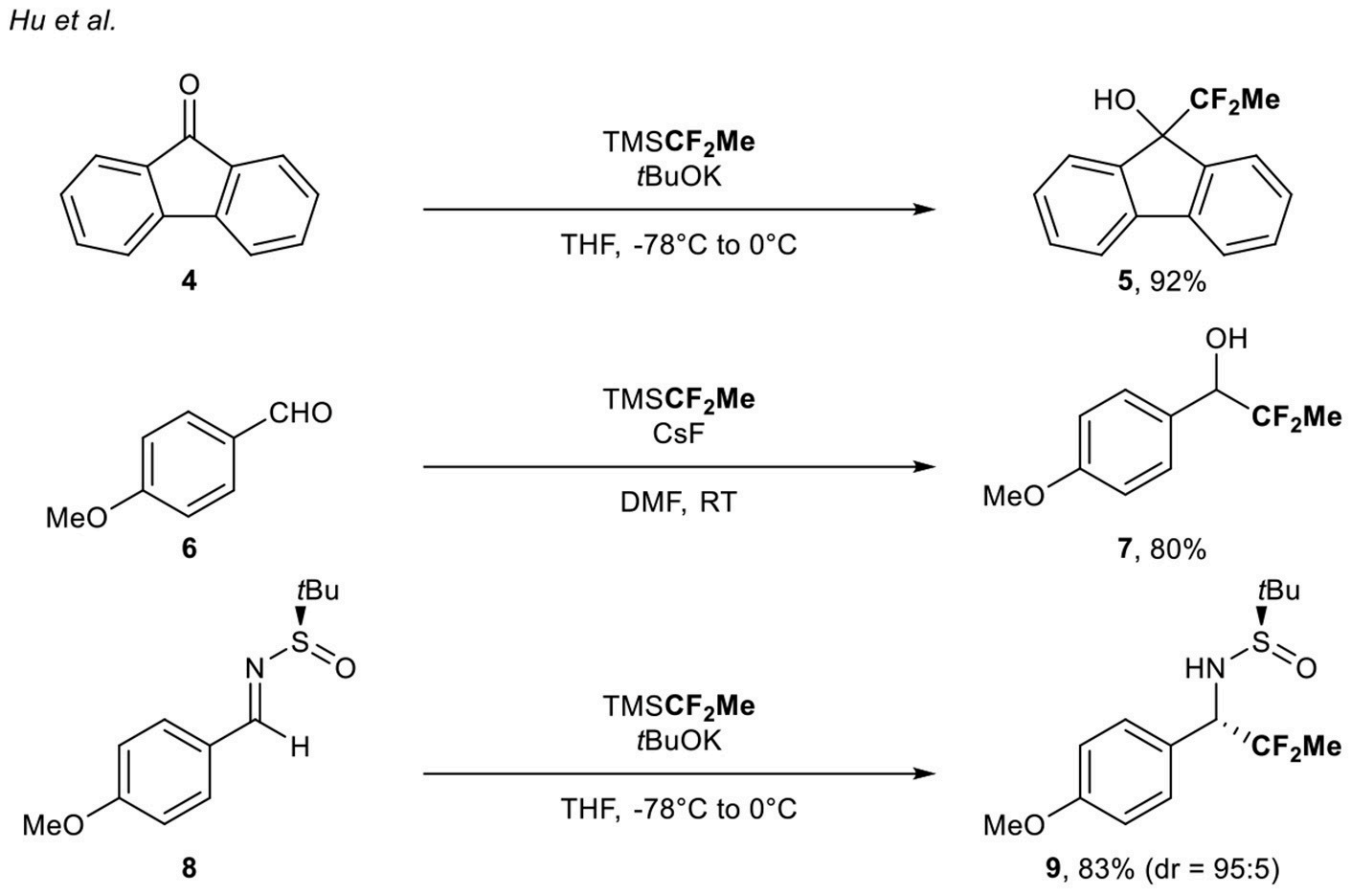
(1,1-Difluoroethyl)trimethylsilane as **CF**_**2**_**Me** source for the functionalization of carbonyl derivatives.

In the course of their studies regarding the difluoromethylation of aldehydes, ketones, and *N-tert*-butylsulfinimines using the nucleophilic TMSCF_2_H source, Hu and co-workers (Zhao et al., 2011) reported the 1,1-difluoroethylation of carbonyl derivatives **4**, **6**, and **8** (three examples, up to 92% yield) with the corresponding (1,1-difluoroethyl)trimethylsilane (TMSCF_2_Me) under basic conditions (Scheme 2).

#### Use of a CF_2_Me-Substituted Phosphonium Salt

**Scheme 3:**
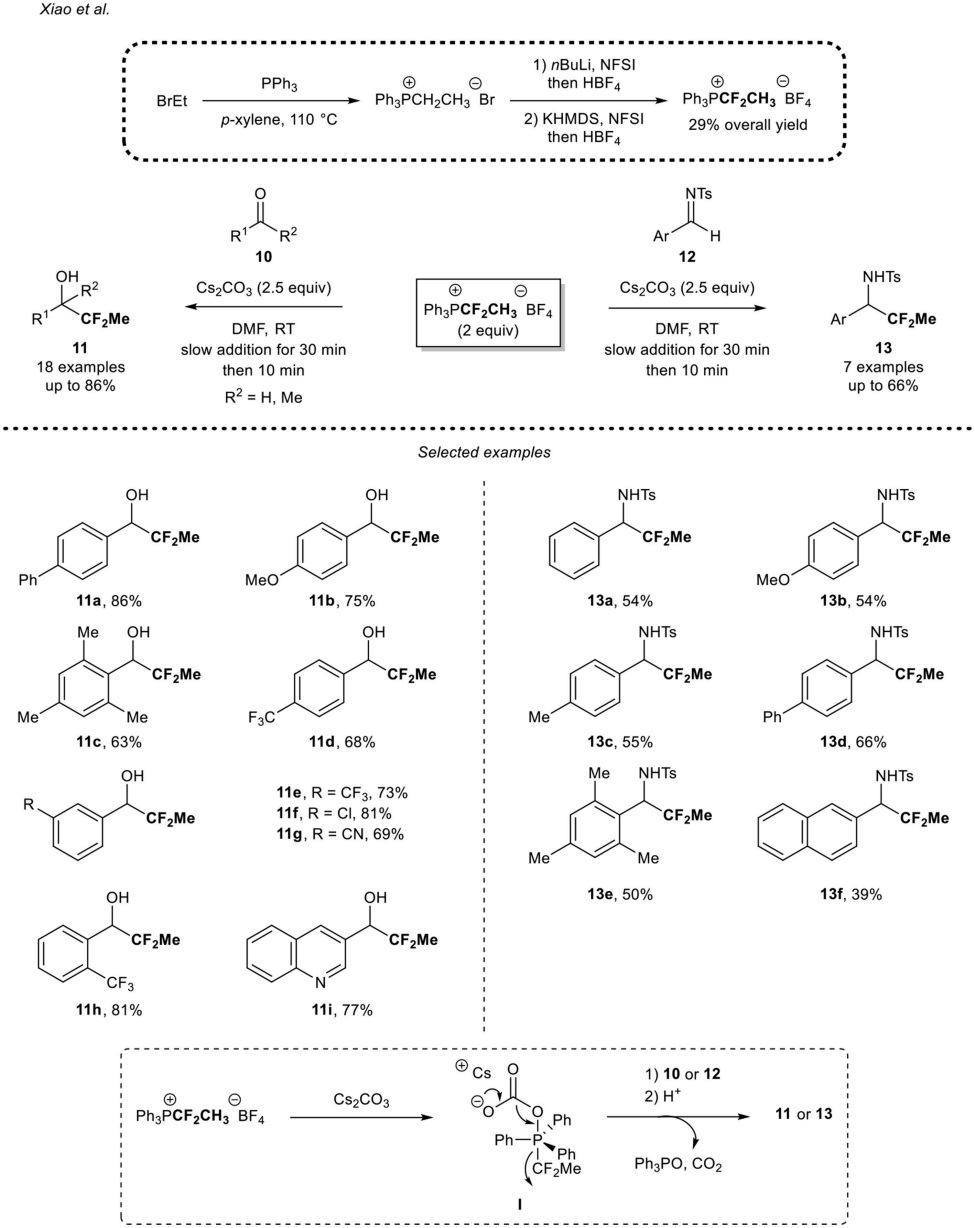
Application of the 1,1-difluoroethyl phosphonium salt for the 1,1-difluoroethylation reaction of carbonyl derivatives.

About 10 years later, Xiao and co-workers reported a new methodology to access CF_2_Me-containing molecules by means of a nucleophilic source (Deng et al., 2016). As an alternative to the powerful TESCF_2_Me reagent, which has a low boiling point and which required a tedious multi-step synthesis [four steps from thiophenol (Mogi et al., 2007)], Lin and Xiao designed a nucleophilic reagent based on a phosphonium salt. The shelf-stable 1,1-difluoroethyl phosphonium salt was prepared in three-steps from the commercially available and inexpensive PPh_3_ and EtBr, in a 29% overall yield (Scheme 3). After the formation of the ethyl phosphonium salt, a subsequent two-step fluorination sequence with NFSI, allowed the formation of the desired reagent on a gram-scale.

This reagent was then successfully applied for the 1,1-difluoroethylation of (het)aromatic aldehydes and the 4-phenyl acetophenone **10**. Electron-rich aryl aldehydes were smoothly converted into the desired secondary alcohols in moderate to high yields, as illustrated with the *para*-phenyl derivative **11a** and the *para*-methoxy derivative **11b**. A slight decrease of yield was observed when the reaction was performed from the sterically hindered electron-rich aldehyde **10c**. Aryl aldehydes bearing an electron-withdrawing substituent (i.e., CF_3_, CN, halogens) were also suitable substrates **10d-h** for the reaction. In addition, the substitution pattern had no impact on the reaction outcome since the *para*-, *meta*-, and *ortho*-CF_3_ substituted aryl alcohols **11d,e,h** were obtained in good yields (68, 73, and 81%, respectively). Note that the 3-quinolinecarboxaldehyde **10i** was functionalized in a good yield. However, only traces of the desired 1,1-difluoroethylated product were detected when starting from an aliphatic aldehyde and a lower ^19^F NMR yield (21%) was observed when the reaction was carried out with 4-phenyl acetophenone. To further demonstrate the potential of the methodology, the authors applied their methodology to the functionalization of the *N*-tosyl imines **12a-f** and the corresponding products **13** were obtained in moderate to good yields (39–66%). It is worth mentioning that due to the lower reactivity of *N*-tosyl imines **12** compared to aldehydes **10**, 1,1-difluoroethylated amines **13** were obtained in lower yields than 1,1-difluoroethylated alcohols **11**. A plausible mechanism was suggested by the authors. After reaction of the Cs_2_CO_3_ promoter with the 1,1-difluoroethyl phosphonium salt, the intermediate **I** would be formed. This latter might then undergo a decarboxylation reaction to afford Ph_3_PO and release a nucleophilic CF_2_Me residue from the cleavage of the P–CF_2_ bond. Then, this species might react with **10** or **12** to afford the desired 1,1-difluoroethyated compound **11** or **13**.

### Transition Metal-Promoted Reactions to Access CF_2_Me-Containing Derivatives

As a complementary strategy, the development of transition metal promoted 1,1-difluoroethylation of molecules offered an efficient synthetic pathway to build up C-CF_2_Me bonds.

#### Access to 1,1-Difluoroethylated Derivatives From Organozinc Reagents

**Scheme 4:**
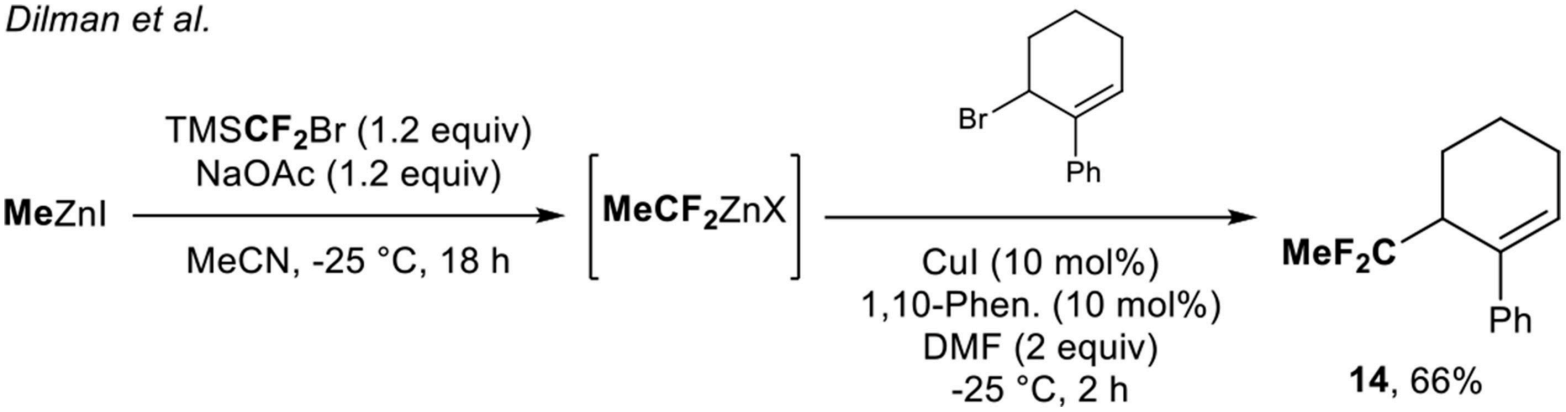
Access to the CF_2_Me-containing molecule **14** *via* a two-step process. 1,10-Phen. = 1,10-Phenanthroline.

The group of Dilman (Zemtsov et al., 2014) depicted a two-step process for the synthesis of 1,1-difluoroethylated derivatives, thanks to the *in situ* formation of the MeCF_2_ZnX species. The reaction of the difluorocarbene with MeZnI formed the desired organozinc reagent. The latter was engaged in a copper-catalyzed allylation reaction and allowed the synthesis of the corresponding CF_2_-containing molecules. With this methodology, a single example of a CF_2_Me-containing molecule **14** was prepared in 66% yield (Scheme 4).

#### Copper-Mediated 1,1-Difluoroethylation Reaction Using TMSCF_2_Me as a Fluorinated Source

**Scheme 5:**
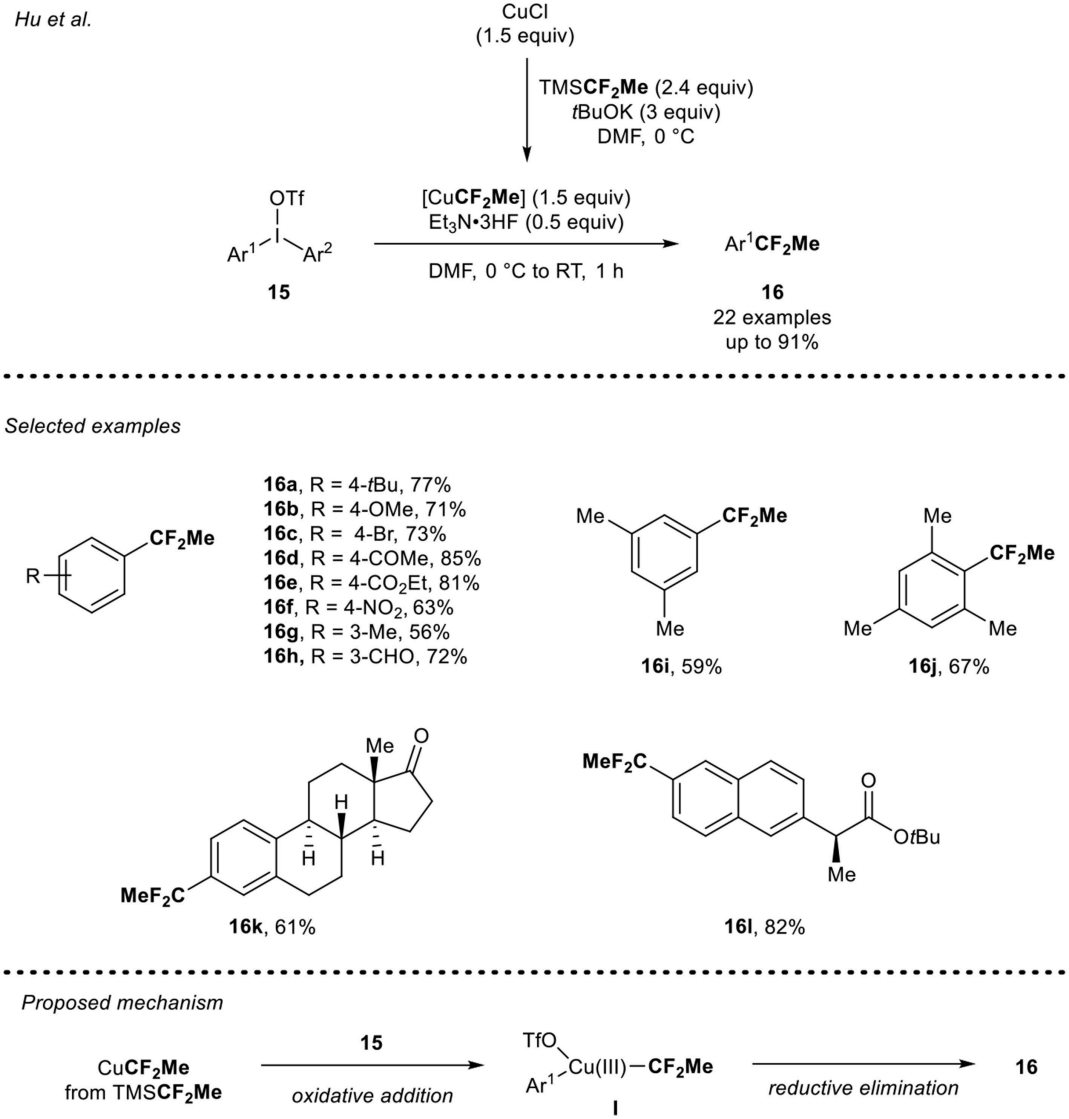
1,1-Difluoroethylation of diaryliodonium salts **15** using the *in situ* generated CuCF_2_Me reagent.

In 2016, Hu and co-workers (Li et al., 2016) described the copper-mediated 1,1-difluoroethylation reaction of diaryliodoniums triflate **15** using CuCF_2_Me (Scheme 5). The CuCF_2_Me reagent was *in situ* generated from the reaction of CuCl with TMSCF_2_Me (Mogi et al., 2007) in the presence of *t*BuOK. Then, the synthesis of (1,1-difluoroethyl)arenes **16** was carried out. In presence of 1.5 equivalents of the *in-situ* generated CuCF_2_Me and 0.5 equivalents of Et_3_N·3HF, as a crucial additive, a panel of electron-rich diaryliodoniums salts were smoothly converted into the desired CF_2_Me-containing arenes **16a,b,g** in good yields. The reaction conditions were also tolerant toward diaryliodoniums salts bearing an electron-withdrawing group such as halogen, ketone, ester, aldehyde, and nitro groups to furnish the 1,1-difluoroethylated arenes **16c-f,h** in moderate to high yields. Sterically hindered diaryliodonium salts **15i** and **15j** were also suitable substrates under these reaction conditions and furnished the desired products **16i-j** in good yields. The potential of this strategy was further demonstrated by the 1,1-difluoroethylation of analogs of relevant compounds such as estrone **15k** and the anti-inflammatory drug naproxen **15l**. Concerning the mechanism, the authors ruled out a radical pathway and proposed the following mechanism pathway: formation of the intermediate Cu(III) species **I**, which would result from the oxidative addition of the CuCF_2_Me species with the diaryliodonium salt **15**, followed by a final reductive elimination step to furnish the expected product **16**.

#### Cobalt-Catalyzed 1,1-Difluoroethylation Reaction of Aryl Grignard Reagent Using BrCF_2_Me

**Scheme 6:**
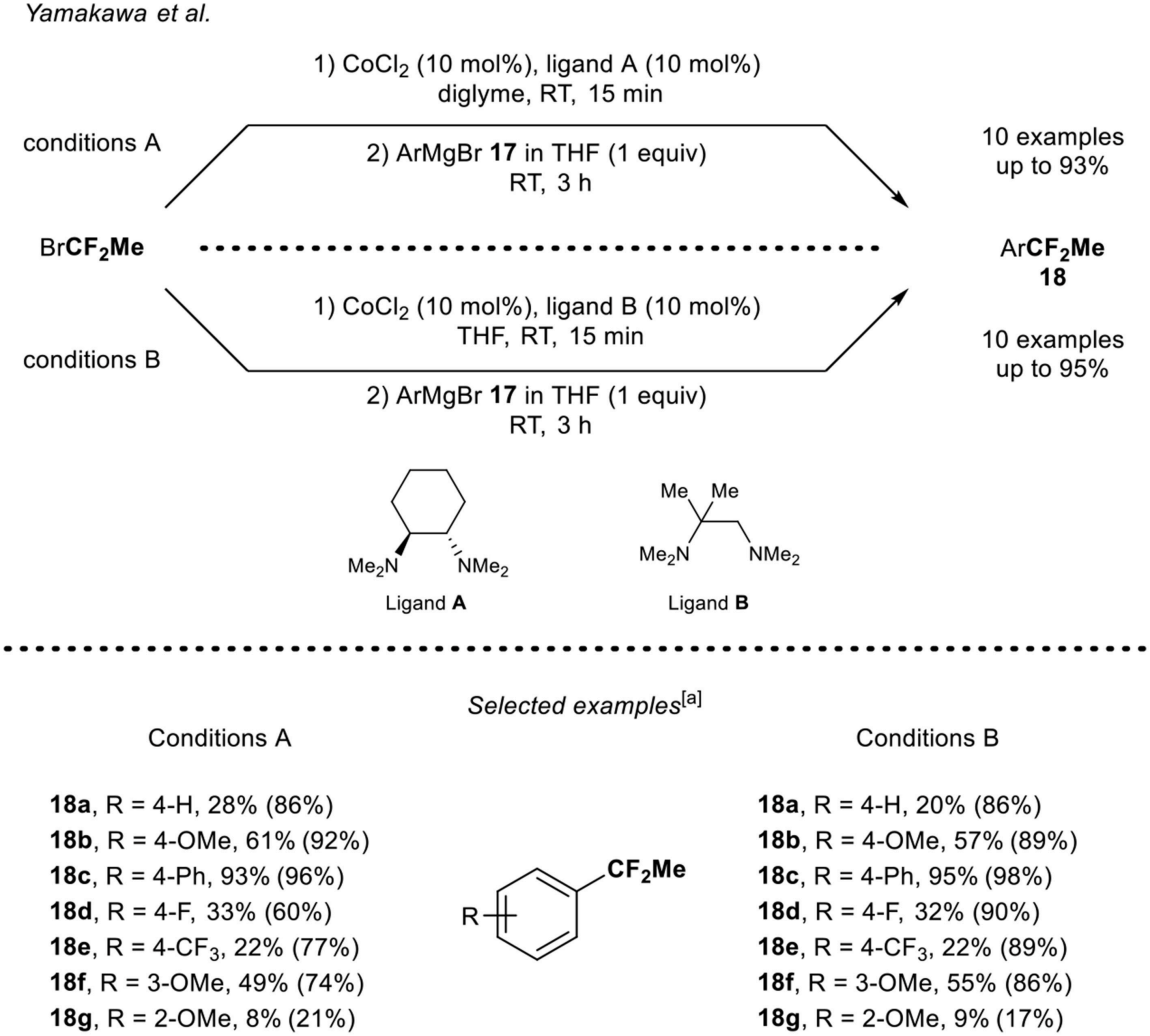
Cobalt-catalyzed 1,1-difluoroethylation of ArMgBr **17** with BrCF_2_Me. [a] Yields shown in parenthesis were determined by ^19^F NMR.

A complementary synthetic route toward the synthesis of 1,1-difluoroethylated arenes was reported by Yamakawa and Ohtsuka (Ohtsuka and Yamakawa, 2016). The cobalt-catalyzed 1,1-difluoroethylation of aryl Grignard derivatives **17a-g** using BrCF_2_Me was developed (Scheme 6). Various aryl Grignard derivatives were functionalized using two sets of reaction conditions. Note that the nature of the ligand and the solvent played an important role in each catalytic system. Aryl Grignard reagents bearing electron-donating and electron-withdrawing groups at the *para* position were functionalized leading to the corresponding products **18** in low to good yields. It turned out that the substitution pattern had an impact on the reaction outcome since the derivative **18g** bearing a methoxy group at the *ortho*-position was obtained in a lower yield compared to the compound **18b** and **18f** with both reaction conditions (A and B). Note that, except for product **18c**, the isolated yields were rather lower than the ones determined by ^19^F NMR, presumably due to volatility issue of the fluorinated products.

## Direct Introduction of the CF_2_Me Moiety via a Radical Process

In this section, the recent breakthroughs for the direct and selective incorporation of the CF_2_Me residue onto molecules via a radical process will be discussed. Note that this strategy was mainly used to access 1,1-difluoroethyl-containing heteroarenes.

### DFES-Na as the 1,1-Difluoroethyl Source

**Scheme 7:**
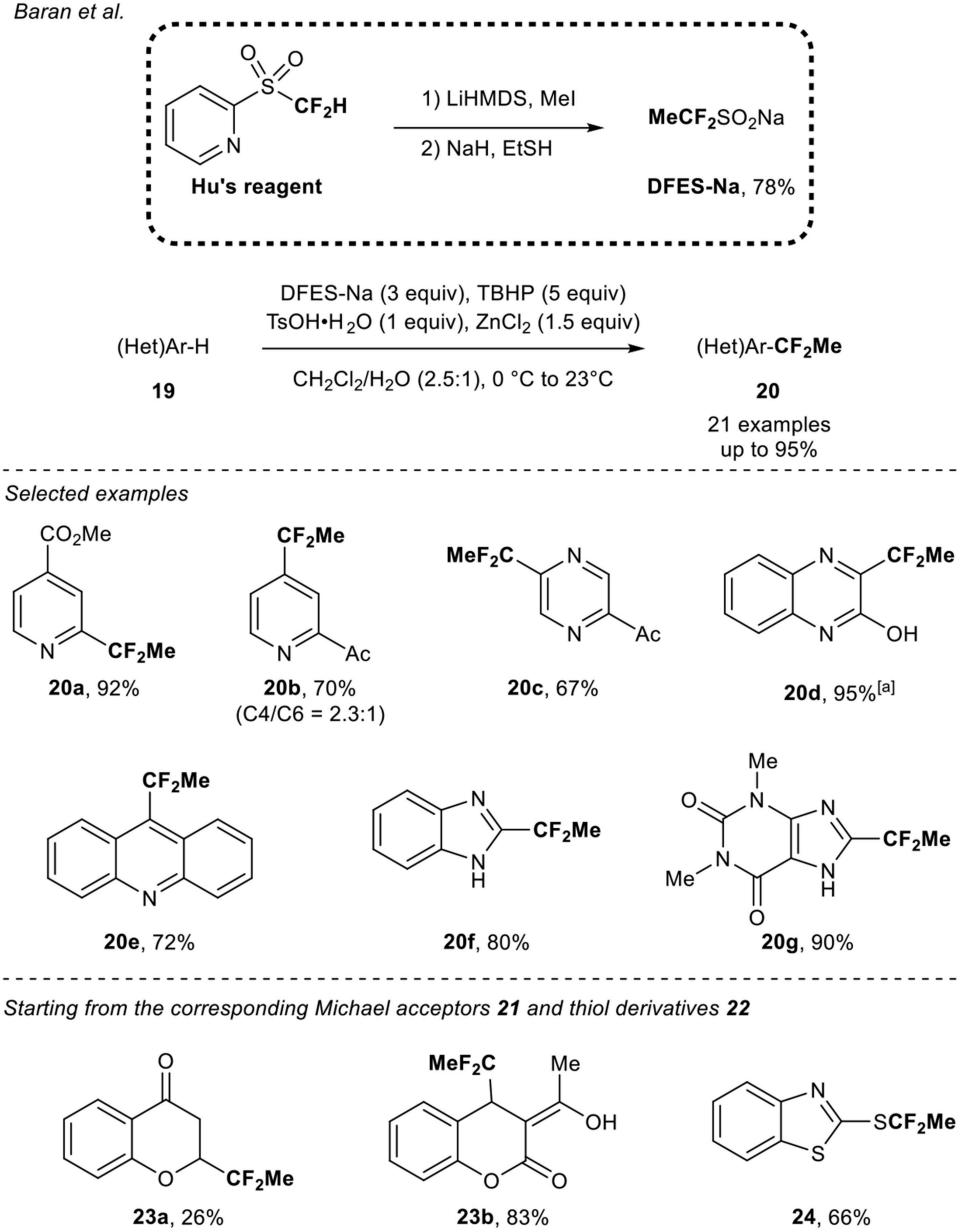
1,1-Difluoroethylation of heteroarenes and extension to other classes of compounds with the DFES-Na reagent. [a] After 10 h using DFES-Na (2 equiv) and ZnCl_2_ (1 equiv).

In 2013, a pioneer work was reported by the group of Baran (Zhou et al., 2013). They designed the synthesis of the sodium difluoroethylsulfinate (DFES-Na) and investigated its application to the functionalization of various heterocycles. The DFES-Na reagent was obtained in two steps from the Hu's reagent and was prepared on a large scale (>100 g). Under oxidative and robust conditions (TBHP, water as co-solvent under air), various classes of heteroarenes **19** were functionalized (21 examples) in the presence of ZnCl_2_ and TsOH^.^H_2_O. The transformation turned out to be functional group tolerant and moderate to good selectivity was observed. In addition, the authors demonstrated the possible direct radical functionalization of Michael acceptors **21** and thiol derivatives **22** (Scheme 7).

**Scheme 8:**
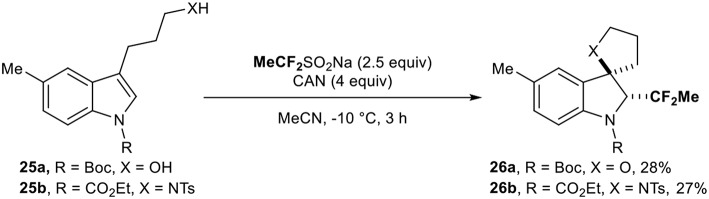
Access to 1,1-difluoroethylated spirocyclic indolines with the DFES-Na reagent.

This reagent was then used by the group of Vincent (Ryzhakov et al., 2017) for the direct introduction of the CF_2_Me residue on protected indoles **25**. The reaction led to the corresponding fluorinated spirocyclic indolines **26** under oxidative conditions (2 examples, Scheme 8).

### CF_2_Me-Containing Sulfones as the 1,1-Difluoroethyl Source

**Scheme 9:**
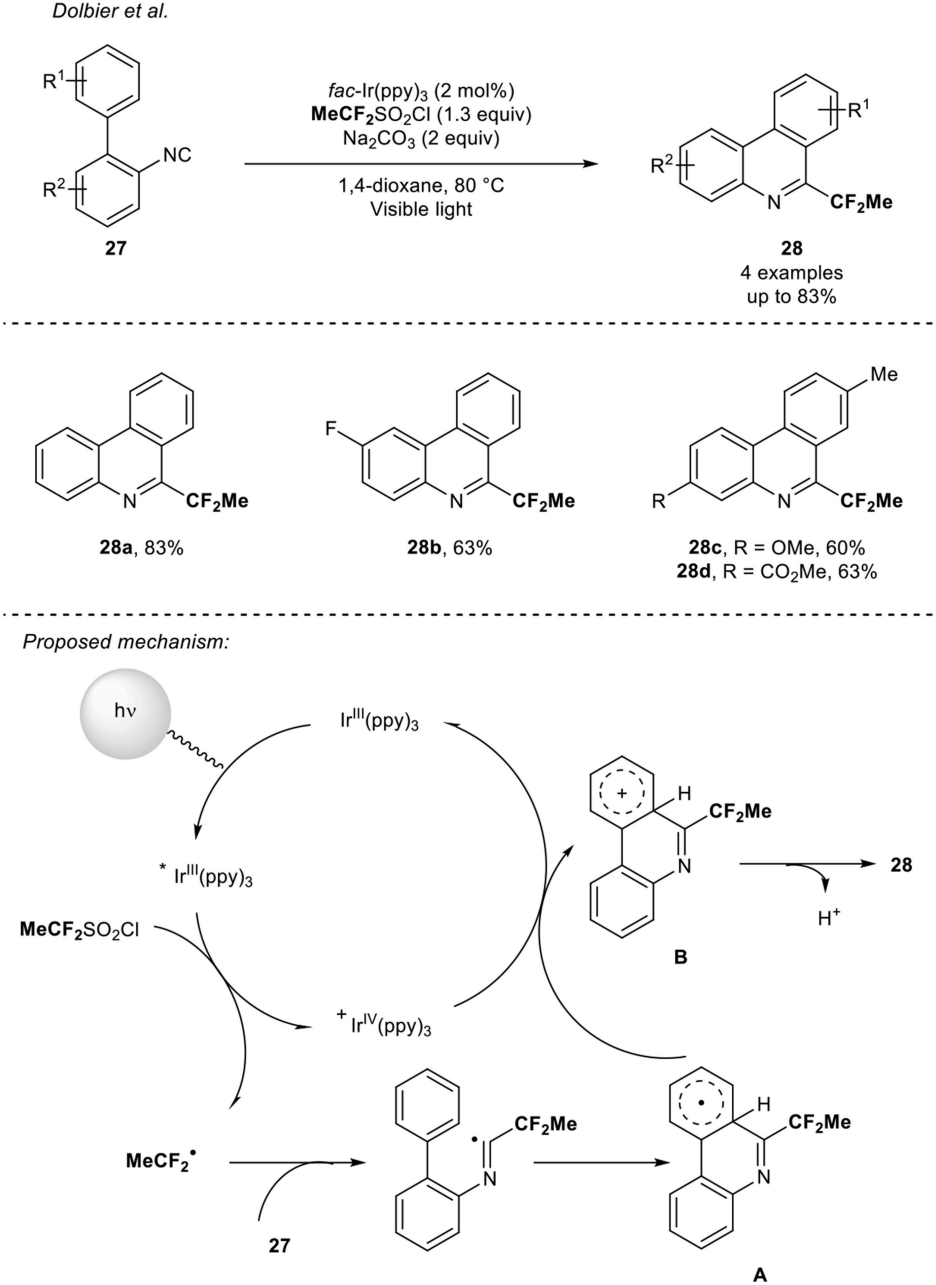
1,1-Difluoroethylation of isocyanides with the reagent **MeCF**_**2**_SO_2_Cl.

In 2015, the group of Dolbier (Zhang et al., 2015) reported the synthesis of fluorinated phenanthridines **28** under visible light photoredox catalysis. Starting from biphenyl isocyanides **27**, the 1,1-difluoroalkylation occurred using an Ir photocatalyst *via* a tandem addition/cyclization/oxidation sequence. Although the study mainly focused on the difluoromethylation reaction, the introduction of the 1,1-difluoroethyl radical was readily performed using the MeCF_2_SO_2_Cl reagent as precursor of the CF_2_Me radical (Scheme 9). Using this reaction manifold, four CF_2_Me-containing phenanthridines were synthesized in good yields (up to 83%). The following mechanism was proposed by the authors: first, the generation of the radical CF_2_Me from the reduction of the MeCF_2_SO_2_Cl reagent with the excited Ir catalyst followed by its addition on the isocyanides **27**. Then a cyclization would lead to the corresponding radical **A**, which would be oxidized into the species **B**, regenerating the Ir-catalyst. A final deprotonation of **B** would yield to the expected products **28**.

**Scheme 10:**
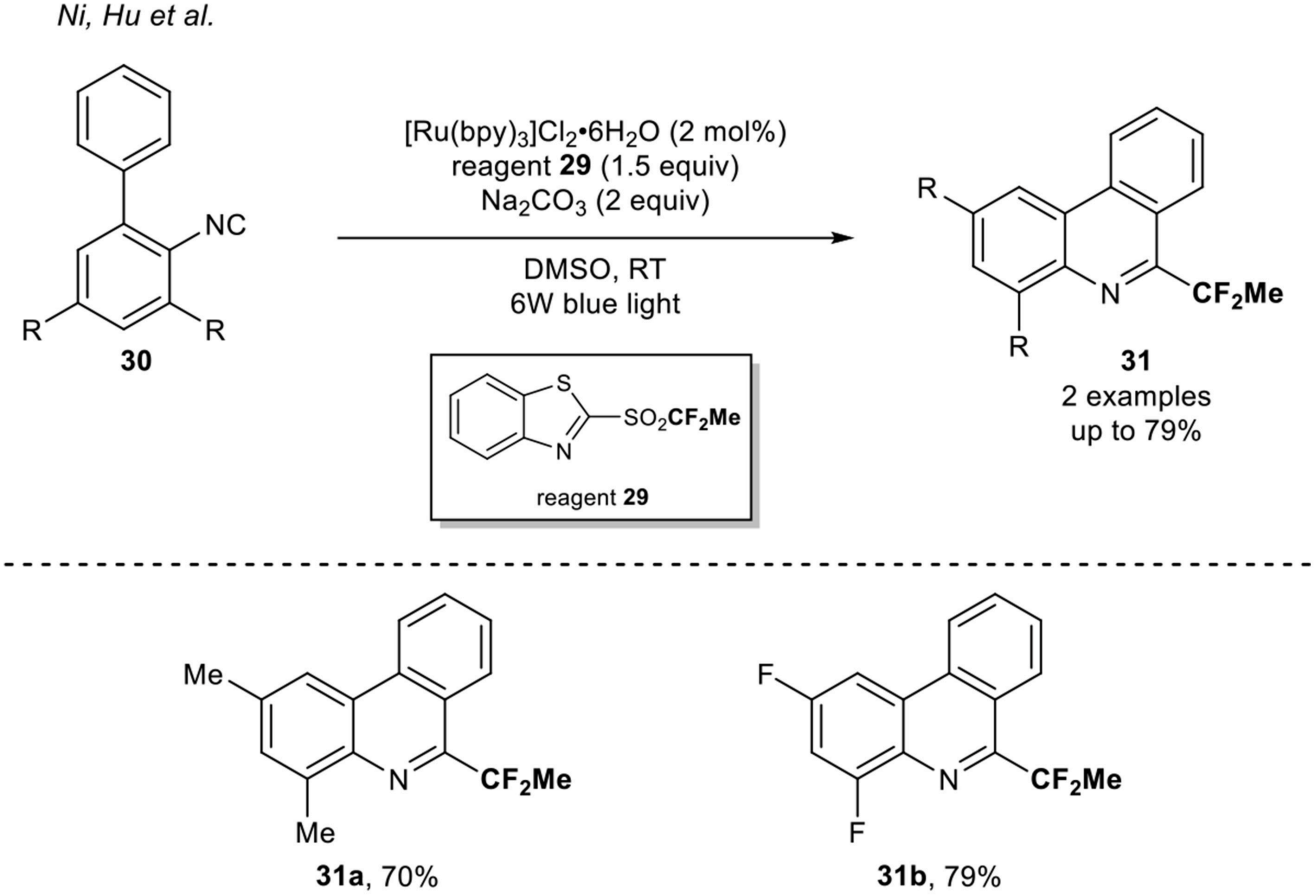
1,1-Difluoroethylation of isocyanides with the reagent **29**.

A complementary approach was reported by Hu and co-workers in 2016 (Rong et al., 2016). They developed a new class of fluoroalkylation reagents, which enabled the formation of R_f_ radical using visible light photoredox catalysis. Using fluoroalkylated heteroaryl sulfones, various isocyanides were functionalized with several fluorinated motifs (R_f_ = CH_2_F, CF_2_H, CF_2_Me, CF_2_Ph, CF_3_, and CF_2_COPh). Among them, the Ru-catalyzed 1,1-difluoroethylation of two biphenyl isocyanides **30** using the reagent **29** was reported (Scheme 10).

**Scheme 11:**
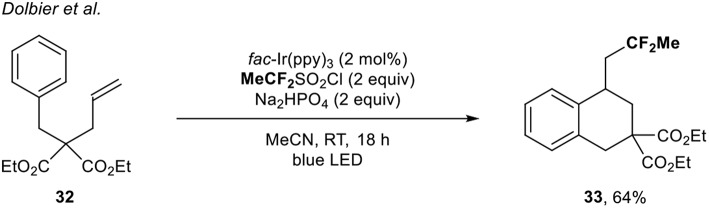
1,1-Difluoroethylation of the unactivated alkene **32** with **MeCF**_**2**_SO_2_Cl.

In 2017, the group of Dolbier (Zhang et al., 2017) developed a radical fluoroalkylation of unactivated alkenes under photoredox catalysis to build up fluorinated tetralin derivatives. In that context, a single example of the direct introduction of the CF_2_Me group to build up the corresponding carbocyclic compound under Ir catalysis was depicted. The expected product **33** was obtained in 64% yield using the MeCF_2_SO_2_Cl reagent (Scheme 11).

## Summary and Outlook

In this Mini-review, we discussed the recent advances made for the direct and selective introduction of the valuable CF_2_Me group. As alternative to the traditional synthetic routes, these direct approaches represented efficient and new retrosynthetic pathways. In this context, technological solutions based on the design of new tools (original strategies and reagents) for the 1,1-difluoroethylation of several classes of compounds were designed. Two strategies were employed based either on the use of nucleophilic reagents or precursors of CF_2_Me radical sources. In the former case, transition metal free approaches as well as transition metal-mediated or -catalyzed transformations were depicted. The complementary synthetic pathway relied on the use of a radical process to access CF_2_Me-containing molecules using carefully designed reagents. Beyond these impressive achievements, further developments for the direct introduction of the CF_2_Me residue, especially for the late-stage functionalization of complex (bioactive) molecules will be of prime importance to enlarge the existing toolbox. Thanks to the pivotal importance of the organofluorine chemistry in various research fields, we strongly believe that this Mini-review will offer new perspectives to further study and apply this original fluorinated moiety.

## Author Contributions

EC and TB collected the literature data related to this review article. EC, TP, PJ, and XP wrote sections of the manuscript. TB wrote the first draft of the manuscript. All authors contributed to the final version of the manuscript and approved the submitted version.

### Conflict of Interest Statement

The authors declare that the research was conducted in the absence of any commercial or financial relationships that could be construed as a potential conflict of interest.
